# Endoscopic retrieval of 195 incidentally found ingested magnets in a pediatric patient: The limitations of radiography

**DOI:** 10.1002/ccr3.8349

**Published:** 2023-12-27

**Authors:** Wissam Jamal Al Tamr, Kareem Omran, Mohammad Aldisi, Hilal Matta, Mohamed Nada

**Affiliations:** ^1^ NMC Royal Hospital Sharjah UAE; ^2^ Department of Public Health and Primary Care University of Cambridge Cambridge UK

**Keywords:** gastroenterology and hepatology, general surgery, oncology, pediatrics and adolescent medicine

## Abstract

Ingestion of magnetic foreign bodies in children can present elusively on radiographs, requiring detailed history for accurate intervention guidance. Clustering and the weight of multiple magnets may indicate falsely distal positions in the GI tract.

## Graphical abstract

Endoscopic view of 195 neodymium magnetic beads in an asymptomatic child's stomach, found incidentally 9 months after ingestion. Localization was impeded by the beads' mass effect, with their removal complicated by the narrowness of the child's esophagus.

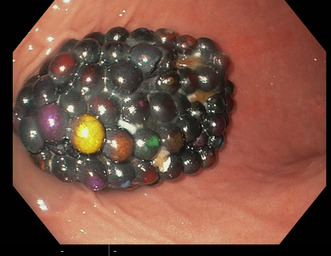

## 1 INTRODUCTION

The ingestion of multiple high‐powered magnets by children represents a serious clinical concern, carrying a substantial risk for significant gastrointestinal injury.^1^ This report discusses a remarkable case involving the ingestion of 195 high‐powered magnets by a child, underscoring the complexities and dangers associated with such incidents. The unique nature of magnet ingestion poses significant risks, including the potential for pressure necrosis, perforation, and fistula formation due to magnets attracting each other across bowel walls.^2^, ^3^, ^4^, ^5^ With the increasing global prevalence of pediatric magnet ingestion and the severe gastrointestinal complications that can arise, prompt, and effective management is crucial for patient safety.^6^

This case exemplifies the diagnostic challenges encountered in pediatric foreign body ingestions, particularly when the cumulative weight of ingested magnets leads to misleading radiographic impressions. Such scenarios can result in a misdiagnosis of the foreign body's location within the gastrointestinal tract, potentially prompting unnecessarily invasive procedures. The successful endoscopic retrieval of this large quantity of magnets not only averted the need for surgical intervention but also highlighted the efficacy of endoscopic techniques in accurately locating and safely extracting ingested foreign bodies, even in substantial numbers. The critical role of vigilant history‐taking becomes evident, especially in light of the limitations of radiography. By detailing this complex case of multiple magnetic bead ingestion and its successful endoscopic management, we aim to shed light on the challenges and strategies essential in handling such intricate pediatric emergencies.

## 2 CASE HISTORY/EXAMINATION

**FIGURE 1:**
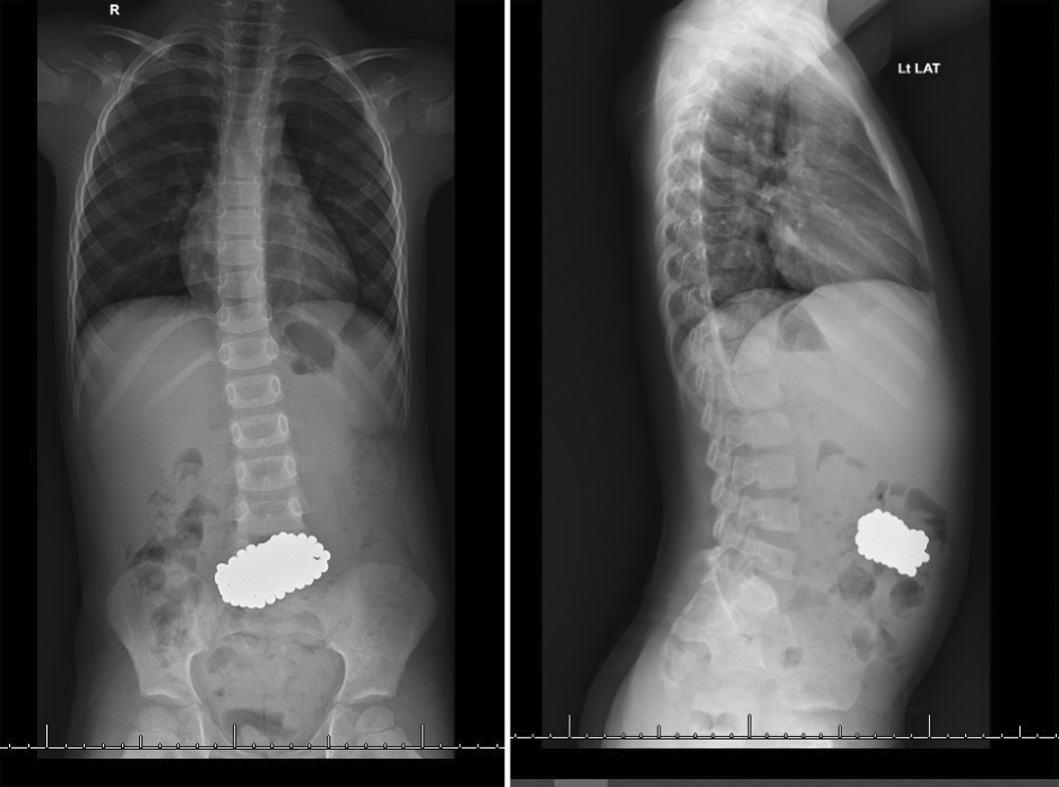
Anteroposterior and lateral radiographs of the abdomen demonstrating a large conglomeration of magnetic beads, appearing as a radio‐opaque cluster, localized in the distal gastrointestinal tract.

A 4‐year‐old male presented to our emergency department following a road traffic accident. He had been admitted to different hospital prior, where had had a CT scan done. However, the patient was discharged by his parents against medical advice, and they presented to this hospital instead. They requested an assessment for potential traumatic injuries resulting from the crash. This included comprehensive clinical examinations, as well as an abdominal X‐ray. An unexpected finding on the abdominal radiograph revealed an aggregation of dense, circular opacities in the mid to lower abdominal region, prompting a differential diagnosis that included foreign body ingestion (Figure 1). Further inquiries into the patient's history from the mother, divulged that magnetic beads purchased 10 months prior, had gone missing for approximately 9 months. She had estimated that they would weigh around 300 g. After thorough consultation and history taking with the patient, he finally admitted that he did indeed swallow the magnets but was scared to admit it. This was done over one sitting, in small quantities in a row. Despite this revelation, the child showed no symptomatic evidence of foreign body ingestion, remaining free from abdominal pain, vomiting, and any gastrointestinal distress both at presentation and in the preceding months. Further comprehensive screening revealed no signs of mental disability, abuse, or maltreatment, affirming the child was well‐cared for and in good health aside from the foreign body incident.

## 3 INVESTIGATIONS

Given the ambiguous localization of the foreign body and the potential for serious complications associated with magnetic objects, a multidisciplinary approach was imperative. Initially, an ultrasound scan was performed, but this did not provide any benefit. A CT scan was proposed as a means to better localize the beads. However, the parents declined this option, citing concerns about redundancy and a desire to minimize their child's exposure to radiation. They were unable to provide the previous CT report due to their earlier decision to discharge against medical advice. After discussions among the pediatric surgery and gastroenterology teams, a stepwise endoscopic intervention was agreed upon since there was uncertainty regarding the location of the foreign object. The initial endoscopic evaluation would be an upper gastrointestinal endoscopy, followed by a colonoscopy if necessary, and a surgical laparotomy would be reserved as a last recourse. This decision process was determined through evaluating the unlikelihood of the large mass crossing the pyloric sphincter, as well as the weight of the object likely falsifying the impression of a more distal location on the radiograph. The decision was also driven by the team prioritizing minimizing the patient's exposure to sedation. Given that a CT scan would necessitate additional sedation, it was decided to limit sedation to the operative theater. This approach meant that if the endoscopy and colonoscopy did not yield definitive results, the patient could undergo a laparotomy under the same general anesthesia, thereby avoiding multiple sedation events. Opting for a CT scan would have introduced an unnecessary extra sedation episode, especially since the management plan would include endoscopy, colonoscopy, or laparotomy regardless of the scan's findings.

## 4 TREATMENT

**FIGURE 2:**
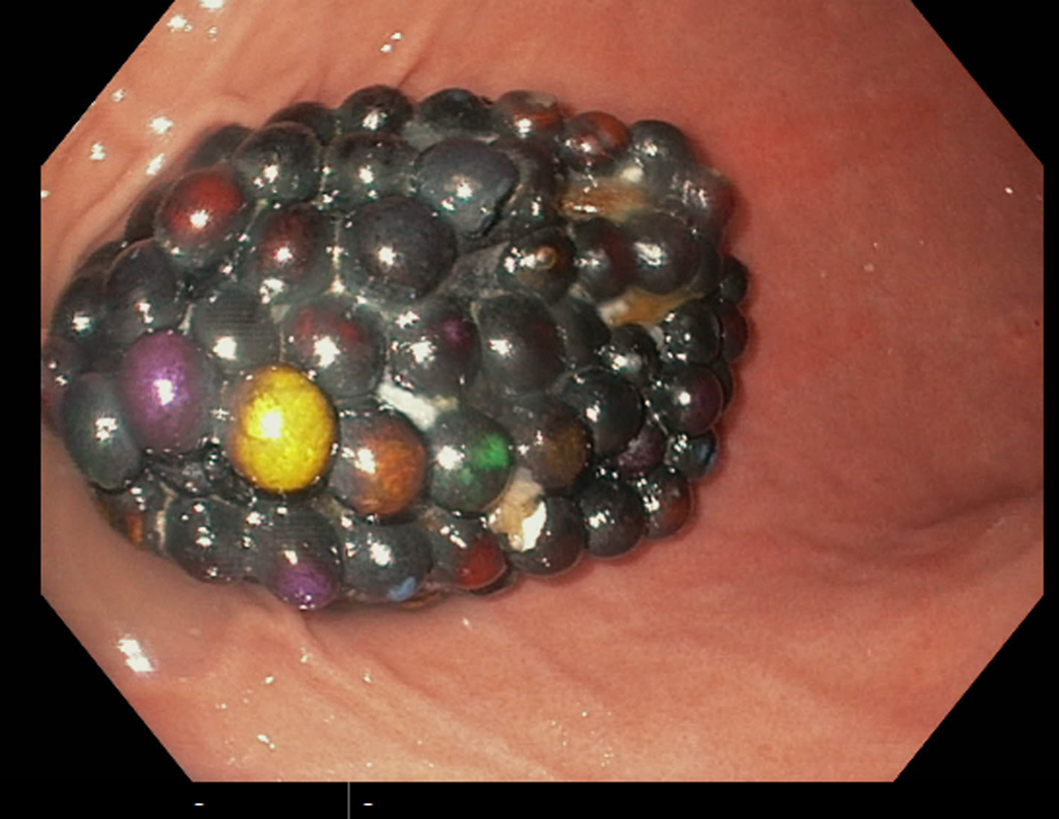
Image captured during upper gastrointestinal endoscopy showing a tightly packed cluster of magnetic beads forming a singular mass.

**FIGURE 3:**
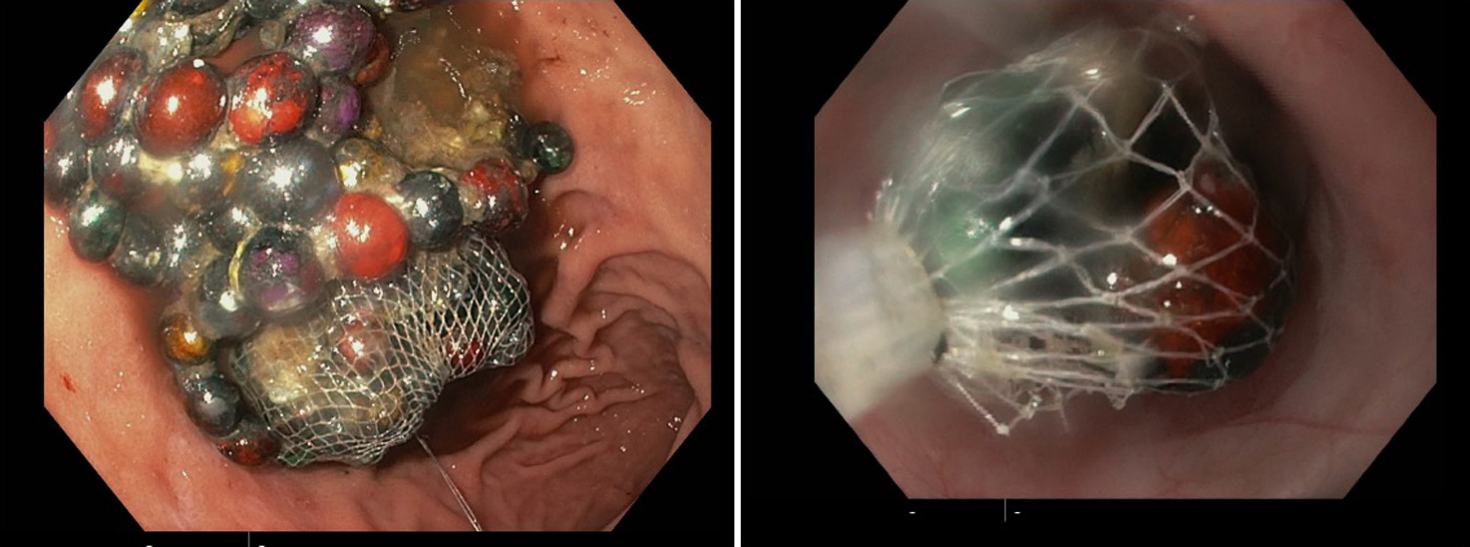
Sequential images demonstrating the use of a net basket technique in upper gastrointestinal endoscopy for the safe retrieval of a clump of magnetic beads from the stomach.

Upper gastrointestinal endoscopy was performed under anesthesia and intubation for tracheal protection (as per local hospital guidelines). The upper gastrointestinal endoscopy was successful and revealed the foreign body residing in the gastric cavity, fortunately without causing any mucosal injury or other immediate complications (Figure 2). The object in question was a conglomerate of magnetic beads as suggested from the history, approximately 10 cm by 8 cm in size, resembling a grape‐like mass. Given the substantial size of the mass relative to the esophageal diameter of 1.5 cm, the extraction posed a significant challenge. The potential for esophageal trauma was averted by employing careful retrieval using an endoscopic net basket technique (Figure 3).

Initially, an attempt was made to extract the beads as a compacted unit. However, this approach was unsuccessful, as the mass was too large to pass through the narrow esophagus. In response to this challenge, the mass was divided into two halves using the net basket. However, due to their magnetic nature, the half within the basket magnetically attracted the half outside, resulting in the same issue as before.

To overcome this, the basket containing one half of the beads was kept in the stomach, and a second basket was employed to carefully separate and retrieve the external half. This technique allowed for the removal of five to nine magnets at a time. The process was carried out sequentially until all magnets were successfully removed, including those initially retained in the original basket.

**FIGURE 4:**
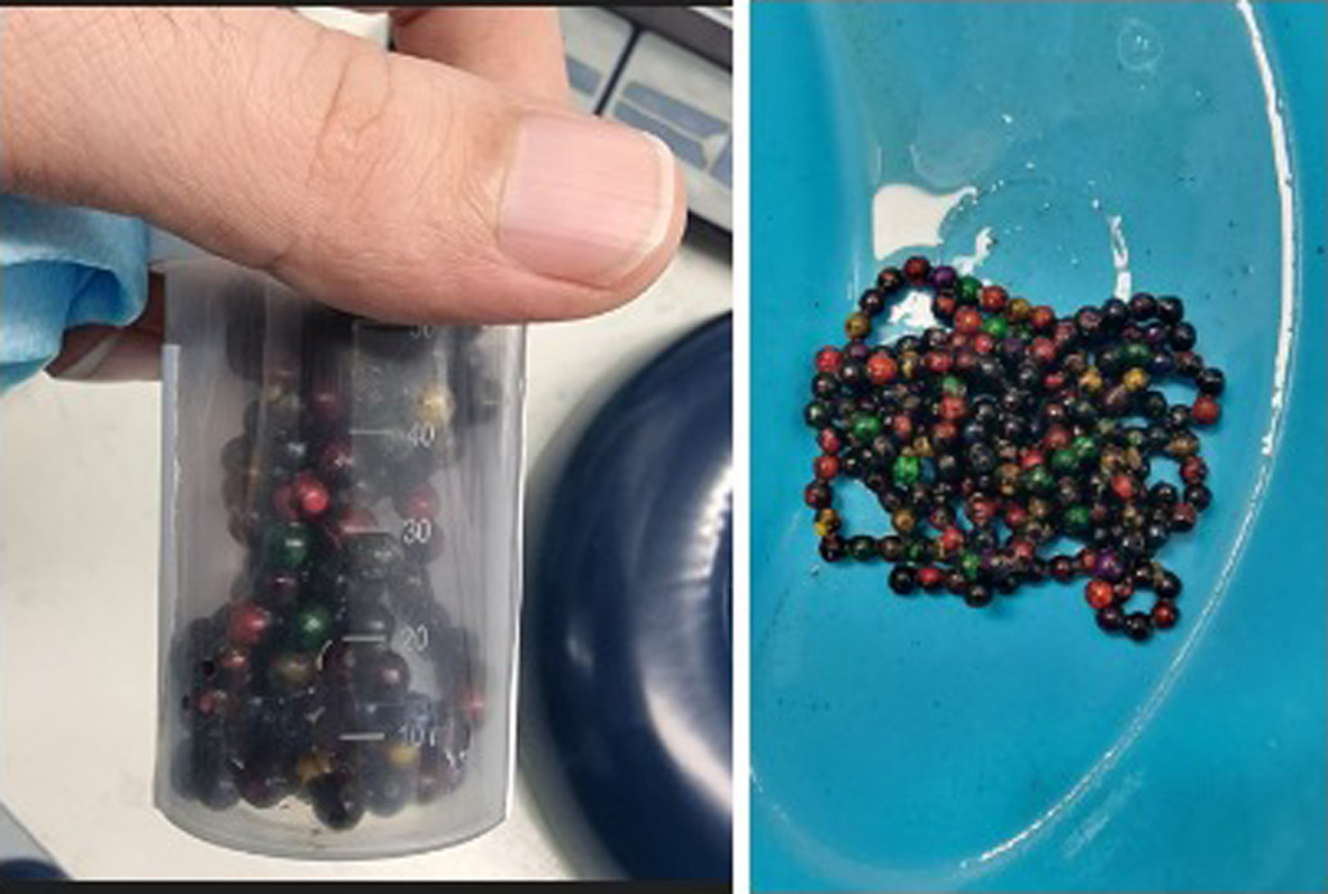
The image on the left shows a container holding the 195 magnetic beads that were endoscopically retrieved from a pediatric patient, while the image on the right displays the beads spread out demonstrating the quantity and varied appearance of the ingested objects.

This method proved successful after an hour of careful manipulation, culminating in the extraction of the entire mass, which was comprised of 195 individual neodymium magnetic beads, weighing approximately 230 g (Figure 4).

To ensure the complete removal of the magnetic objects, the patient underwent both chest and abdominal radiographs immediately post‐procedure. These images confirmed the absence of residual foreign bodies. The patient tolerated the procedure well and was monitored closely for any delayed complications, none of which occurred during his hospital stay.

## 5 DISCUSSION

Ingestion of foreign bodies is a frequent occurrence in pediatric cases, with most instances resolving spontaneously and harmlessly.^7^, ^8^ Yet, the ingestion of several high‐powered magnets notably elevates the risk of adverse health outcomes.^9^, ^10^ To investigate suspected cases of ingestion, an abdominal X‐ray is considered the gold standard for detecting and localizing ingested magnet foreign bodies, as most magnets are radiopaque.^11^, ^12^ However, existing literature highlights certain limitations of diagnostic imaging: both plain X‐rays and computed tomography scans demonstrate restricted sensitivity in ascertaining the actual number of ingested magnet pieces. Specifically, X‐rays may misleadingly depict a single bead‐like magnet as multiple entities, while two closely bound identical pieces could be perceived as a singular object.^7^ This information emphasizes the challenge in accurately assessing the number of ingested magnets based solely on imaging. In this case, radiographs were performed as a part of routine examination post road traffic accident, and incidentally suggested a large single mass radiopaque mass. Only through the history was it determined that this was actually over 100 magnet beads, suggesting the misleading nature radiography may provide without holistic investigations.

Furthermore, this case presents a prime example of the effect of gravity on large mass foreign bodies, and how this affects their localization. The radiographic detection of a foreign body in the mid‐abdominal region, despite its actual presence in the stomach, underscores the role of gravitational influence in displacing such objects. This understanding is vital for correctly utilizing diagnostic tools and managing patients with foreign body ingestions. A key aspect of this case was recognizing that the ingested object was likely heavy, due to being a substantial clump of magnetic beads, deduced from a detailed history provided by the patient's mother. The weight of the magnetic bead conglomerate likely contributed to a downward pull, complicating the interpretation of radiographic data and underscores the need for a comprehensive assessment of an object's physical properties by physicians, prior to attempting invasive investigations. This is further supported by literature recommending endoscopy as the first‐line intervention for non‐pelvic magnet ingestions.^13^ In our case, endoscopic retrieval was justified and aligns with current best practices. The high success rate of endoscopic removal in pediatric foreign body ingestions, up to 98.5%, further validates this approach.^14^, ^15^, ^16^ Our use of the endoscopic net‐basket technique, avoiding surgical intervention, mirrors these findings and is particularly effective for large metallic foreign bodies.^17^

The asymptomatic nature of this case, despite the ingestion of 195 magnetic beads, aligns with reports suggesting that many pediatric foreign body ingestions go unnoticed.^18^ This raises significant concerns about the ease of access and the attractiveness of these magnets to children. They are often small, round, and sold in large quantities as toys, increasing the risk of such incidents. This clinical observation leads us to a broader public health discussion. The frequent occurrence of magnet ingestion cases, driven by their physical characteristics and appeal, necessitates a shift in focus toward prevention and awareness. In many countries, including the United States, legislative efforts and public awareness initiatives have been pivotal in addressing these risks.^19^ The CPSC's directive in 2012, its overturning in 2016, and the recent 2022 regulations reflect the evolving strategies to regulate these products.^20^, ^21^ Despite these efforts, challenges persist, as indicated by the NASPGHAN survey on the ineffectiveness of warning labels.^22^ Furthermore, the rebranding of these magnets as “adult table toys” and their use as fake piercings in adolescents have contributed to an increase in ingestion cases among older children and young adults.^23^ The marked decrease in cases post‐product recalls and new legislation, as reported by Rosenfield et al., underscores the impact and necessity of such measures.^23^

Public health campaigns, like the #SafeFashion campaign in the United Kingdom, and the work of organizations like the Child Accident Prevention Trust and the Royal Society for the Prevention of Accidents are crucial in raising awareness about the risks of magnet ingestion. The establishment of registries, such as the MAGNETIC study in the United Kingdom and the National Poison Centre in the United States, is vital for collecting data to inform best practices and legislative actions.

## 6 CONCLUSION

This case of a pediatric patient ingesting 195 magnets and their successful endoscopic retrieval emphasizes the importance of vigilant history‐taking and the limitations of radiography in large magnetic foreign body ingestions. Radiographic imaging, while useful, may not always accurately localize dense, aggregated objects such as magnets and may suggest falsely distal locations, resulting in unnecessary invasive procedures. This case also highlights the often‐asymptomatic nature of pediatric foreign body ingestions, with the extremely unusual scenario of 195 magnets being undetected for over 9 months. While no complications occurred to this child, removal is an urgent necessity due to the risks associated. The net basket endoscopic technique, by facilitating a minimally invasive yet highly effective removal, provides valuable insights for clinicians managing similar pediatric emergencies. Moreover, this case serves as a crucial reminder of the need for increased public health awareness and proactive preventive measures, particularly in educating caregivers and regulating magnetic toy sales, to mitigate the risks associated with such dangerous ingestions.

## AUTHOR CONTRIBUTIONS

**Wissam Jamal Al Tamr:** Conceptualization; investigation; methodology; project administration; supervision; validation; visualization; writing – original draft; writing – review and editing. **Kareem Omran:** Conceptualization; investigation; methodology; resources; writing – original draft; writing – review and editing. **Mohammad Aldisi:** Conceptualization; data curation; investigation; methodology; visualization; writing – review and editing. **Hilal Matta:** Conceptualization; data curation; methodology; project administration; supervision; validation; writing – review and editing. **Mohamed Nada:** Conceptualization; data curation; formal analysis; investigation; methodology; supervision; validation; writing – review and editing.

## CONSENT

Written informed consent was obtained from the patient to publish this report in accordance with the journal's patient consent policy.

## Data Availability

Data sharing not applicable to this article as no datasets were generated or analysed during the current study.
